# Modulatory Effect of Monochromatic Blue Light on Heat Stress Response in Commercial Broilers

**DOI:** 10.1155/2017/1351945

**Published:** 2017-06-18

**Authors:** Safaa E. Abdo, Seham El-Kassas, Abeer F. El-Nahas, Shawky Mahmoud

**Affiliations:** ^1^Department of Animal Wealth Development, Faculty of Veterinary Medicine, Kafrelsheikh University, Kafr El-Shaikh, Egypt; ^2^Department of Animal Husbandry and Animal Wealth Development, Faculty of Veterinary Medicine, Alexandria University, Alexandria, Egypt; ^3^Department of Physiology, Faculty of Veterinary Medicine, Kafrelsheikh University, Kafr El-Shaikh, Egypt

## Abstract

In a novel approach, monochromatic blue light was used to investigate its modulatory effect on heat stress biomarkers in two commercial broiler strains (Ross 308 and Cobb 500). At 21 days old, birds were divided into four groups including one group housed in white light, a second group exposed to blue light, a 3rd group exposed to white light + heat stress, and a 4th group exposed to blue light + heat stress. Heat treatment at 33°C lasted for five h for four successive days. Exposure to blue light during heat stress reduced MDA concentration and enhanced SOD and CAT enzyme activities as well as modulated their gene expression. Blue light also reduced the degenerative changes that occurred in the liver tissue as a result of heat stress. It regulated, though variably, liver *HSP70*, *HSP90*, *HSF1*, and *HSF3* gene expression among Ross and Cobb chickens. Moreover, the Cobb strain showed better performance than Ross manifested by a significant reduction of rectal temperature in the case of H + B. Furthermore, a significant linear relationship was found between the lowered rectal temperature and the expression of all HSP genes. Generally, the performance of both strains by most assessed parameters under heat stress is improved when using blue light.

## 1. Introduction

Heat stress is one of the most serious problem facing poultry production in all subtropical countries during summer [1]. The severity of heat stress is due to the resultant oxidative stress which is characterized by accumulation of oxygen reactive species (ROS) in an excess to cellular antioxidants [2–4]. Besides heat exposure, vigorous bird handling, presence of oxidize dietary oils, and infection are associated with ROS formation [3, 5, 6]. ROS accumulation is accompanied by disturbances of cellular balance and modulation of several biological macromolecules including nucleic acid and protein [7]. The cellular antioxidant enzymes represent the first defense system which is responsible for restoring cellular hemostasis. Thus, the increase in the antioxidant enzyme activities including superoxide dismutase (SOD) and catalase (CAT) protects the cells from heat stress-ROS-associated damaged effects. This response greatly differs according to the heat stress conditions, species, and affected tissue [2].

Moreover, one of the main other consequences of heat stress is protein damage and subsequent accumulation of unfolded proteins [8, 9]. Affected cells increase the expression of chaperone proteins and heat shock protein (HSPs), leading to proteostasis and thermotolerance [10]. The HSPs include Hsp40, Hsp60, Hsp70, Hsp90, Hsp110, and the small HSPs. HSP70 and HSP90 are the most conserved HSPs. They work to protect the cell and prevent the aggregation of unfolding protein [11]. Additionally, HSPs protect the cells from heat shock deleterious impacts and enhance tissue repair [12]. HSP expression is regulated mainly at the level of transcription by four heat shock transcription factors (HSFs). HSFs include HSF-1, HSF-2, and HSF-4 (specific to mammals) and HSF-3, which is avian specific [13]. HSFs modify HSP expression through interaction with a specific DNA sequence (heat shock element (HSE)) in their promoter [14, 15]. Hence, they regulate the HS response.

Different approaches have been done to control the destructive effects of heat stress. Among which were inclusion of feed additives in the diet and water, as well as light management [16]. However, lighting management studies in the alleviation of heat stress deleterious effects are still lacking. Previous studies looked at the effect of different monochromatic lights (white, red, green, and blue) on the broiler immune response and the breed performance [17–20]. Light management was found to increase productivity and improve animal welfare [18, 20, 21]. Thus, light color has been considered as a powerful management that can be used to modify many physiological, immunological, and behavioral pathways [22, 23]. For instance, blue light has been shown to have calming effect by reducing the negative impact of different stressors [24, 25]. Blue light modulates peripheral blood T lymphocytes proliferation, the response to Newcastle disease virus vaccine, heterophils to lymphocytes (H/L) ratio, and interleukin-1*β* (IL-1*β*) expression [22, 23]. In addition, using blue light significantly increases the numbers of intestinal intraepithelial lymphocytes, goblet cells, and IgA^+^ cells [26]. Moreover, blue light significantly improves meat quality by decreasing lipid peroxidation and improving antioxidant activities by enhancing SOD, GHS, and total antioxidant capability activities and reduced MDA content both in breast and thigh muscles [27].

The aim of this work was to investigate effects of the monochromatic blue light (BL) on alleviating the negative impact of induced cyclic chronic heat stress in commercial broiler strains. We investigated the regulatory effect of using monochromatic blue light during heat stress on heat stress biomarkers activity including antioxidant enzyme activity, histopathological changes in the liver tissue, HSP gene expression, and bird's temperature.

## 2. Materials and Methods

### 2.1. Bird Management

A total of 160 one-day-old chicks from two commercial broiler strains (Ross 308 and Cobb 500) were used in this experiment. Eighty chicks of mixed sexes were used for each strain. Chicks were purchased from a reputable dealer at El-Gharbia Governorate, Egypt. The birds were housed (10 birds/m^2^) in separate environmentally controlled rooms at the poultry farm, Kafrelsheikh University, Egypt. Each bird had 2.5 cm of feeder space for the first two weeks. After that, 6 cm/bird was allowed. During heat treatment, the feeder and waterer space per bird was increased. The birds were exposed, for the first three weeks, to white light (WL, 400–760 nm) using a light-emitting diode (LED) system. Light duration was adjusted according to the bird's age. Thus, birds received 24 h light length from 0 to 7 days of age and then a light-dark cycle (23 hours : 1 hour) was applied. Light intensity was adjusted according to the Council of the European Communities, 2007 [28, 29]. Thus, birds received 40 lux light intensity for the first week followed by 25 lux till the end of the experiment. Chicks had ad libitum access to feed and water, and diets were formulated to meet the nutrient recommendations for poultry by the National Research Council [30]. Additionally, birds received their regular vaccination program which consisted of Newcastle disease vaccine at days 7 and 18 of age as well as Gumboro disease vaccine at the 14th day of age. The bird's management procedures were undertaken in accordance with the requirements of the Animal Care and Ethics Committee of the Faculty of Veterinary Medicine, Kafrelsheikh University, Egypt.

### 2.2. Experimental Design

At 21 days of age, birds were individually weighed. All birds were divided into equal eight groups (four groups for each strain (*n* = 20) (Table 1). Two groups (W and B) were housed at normal temperature of 24°C with white and blue lighting, respectively. H + W and H + B groups were exposed to an experimental cyclic chronic heat stress. In this regard, heat treatment extended for four successive days in which birds were exposed each day to 33 ± 2°C for 5 h and then the temperature was decreased to normal 24°C for the rest of the day [31]. In the case of the H + B group, white light was replaced with a monochromatic blue light (480 nm, intensity 25 lux). Air humidity was kept at 70% during the experimental period.

### 2.3. Sample Collection

Seven birds from each group were used for the sample collection immediately at the end of the heat treatment (at the end of fourth day heat treatment). The birds were killed by cervical dislocation. Three liver specimens were collected from each bird. One liver specimen was collected in 10% formalin for histopathological examination; the second specimen was collected in PBS and kept at −20°C for analysis of antioxidant enzymes activity while the third liver specimen was placed in 2 ml Eppendorf and immediately frozen in liquid nitrogen then kept at −80°C for RNA extraction.

### 2.4. Histopathological Examination

Liver tissue specimens were fixed in 10% neutral buffered formalin for 24 hours. Then, the tissue was routinely processed in paraffin. Sections (4 *μ*m) from each specimen were obtained from each block and mounted on a glass slide. Tissue sections were subsequently stained with hematoxylin and eosin (H&E) according to the method described by [32]. Liver sections were then examined using a light microscope (200x).

### 2.5. Assays for Measurement of Malondialdehyde (MDA) Content and Antioxidant Enzyme Activities

Liver tissue specimens were ground in sterile cold potassium phosphate buffer (pH 7). Liver homogenates were spun down at 4000 rpm for 15 min at 4°C, and supernatant was used for further assessments. For malondialdehyde (MDA), its concentration was measured using Biodiagnostic kit following the manufacture's protocol (Biodiagnostic, # MD 2529, Egypt). MDA content were measured using UV-Vis spectrophotometer at 534 nm. MDA content was determined as nmol/g of tissue. Superoxide dismutase activity (SOD) was measured following the protocol of Biodiagnostic kit (Biodiagnostic, # SD 2521, Egypt). The change in absorbance at 560 nm over 5 min was measured using UV-Vis spectrophotometer. Additionally, catalase (CAT) activity was measured based on the spectrophotometric method described by [33]. Catalase reacts with a known quantity of hydrogen peroxide, and the reaction is stopped after 1 min with catalase inhibitor. In the presence of peroxidase, the remaining hydrogen peroxide reacts with 3,5-dichloro-2-hydroxybenzene sulfonic acid and 4-aminophenazone to form a chromophore with a color intensity inversely proportional to the amount of catalase in the sample. The absorbance was measured at 240 nm over 3 min. Enzyme activities of SOD and CAT were measured as units/gram of tissue (u/g).

### 2.6. RNA Isolation and Reverse Transcription

Total RNA was extracted from 30 to 50 mg of liver tissue (*n* = 3 from each group) using the TRI reagent (easy-RED™, iNtRON Biotechnology), according to the manufacturer's protocol. The integrity of the RNA was verified by gel electrophoresis through visual inspection of rRNA bands (18S and 28S) in ethidium bromide-stained 2% agarose. Also, RNA concentration was measured by Nanodrop ND1000 (UV-Vis spectrophotometer Q5000/USA). Two *μ*g of RNA sample was reverse transcribed using the SensiFAST™ cDNA synthesis kit (Bioline, United Kingdom). The cDNA product was verified by conventional PCR using HSP70 primers and analyzed by agarose gel electrophoresis.

### 2.7. Real-Time PCR

For gene expression of *HSP70*, *HSP90*, *HSF1*, *HSF3*, *SOD*, and *CAT*, specific primers (Table 2) were used to amplify gene products. In this regard, a real-time PCR (qPCR) was performed using the SensiFAST SYBR Lo-Rox kit (Bioline, United Kingdom) and PikoReal™ 24 Real-Time PCR System (PikoReal 24, Thermoscientific, TCR0024). The reaction mix consisted of 10 *μ*l of SensiFAST SYBR Lo-Rox mastermix, 0.5 *μ*M of each prime, and 2 *μ*l of cDNA. The thermal cycling conditions were initial denaturation at 95°C for 15 min, followed by 40 cycles at 95°C for 15 s, and annealing for 1 min at 60°C for all genes. Dissociation curve analyses were performed beginning at 65°C and ending at 95°C, with incremental increases of 0.5°C every 5 s to validate the specificity of the PCR products. For all tested genes, dissociation curve analysis showed only one peak at the specific melting temperature (data not shown), showing that the PCR products were specifically amplified. All genes were tested in duplicates for three birds of each chicken strain. CT values for each sample were determined and incorporated in “fold change” calculation based on the Livac method [34], and mRNA expressions for each sample were normalized against *β-*actin and GAPDH.

### 2.8. Statistical Analysis

Statistical analysis of the data was performed using GraphPad Prism 6 software (GraphPrism Software, La Jolla, California, USA). Two-way ANOVA followed by Fisher's LSD was used to examine the statistically significant differences of the strain and light treatment effects on heat shock parameters measured including SOD, CAT MDA, and gene expression of *SOD*, *CAT*, *HSP70*, *HSP90*, *HSF1*, and *HSF3* as well as bird's body temperature. Linear regression analysis was performed to determine the association between enzyme activity of SOD and CAT and their gene expressions (*SOD* and *CAT* genes). Additionally, the relationship between the bird's body temperature and *HSP70*, *HSP90*, *HSF1*, and *HSF3* gene expression during heat stress (H + W and H + B) was assessed using linear regression analysis. The results were stated as mean ± SEM. Differences were considered to be statistically significant at *p* values <0.05^∗^, *p* < 0.01^∗∗^, *p* < 0.001^∗∗∗^, and *p* < 0.0001^∗∗∗∗^. The significant difference in the case of regression analysis was determined at *p* < 0.1^∗^, *p* < 0.05^∗∗^, and *p* < 0.01^∗∗^.

## 3. Results and Discussion

### 3.1. Blue Light Significantly Increases Antioxidant Enzyme Activities and Lowers MDA Concentration in Chicken Liver

The analysis of antioxidant enzyme activities as well as MDA content has been considered as one of the most interesting and promising approaches in this study; we reasoned that blue light management would help in treating and preventing of oxidative damage caused by heat stress. Herein, we measured MDA content and SOD as well as CAT enzyme activities in the liver tissue of Ross 308 and Cobb 500 chickens (*n* = 7) after a cyclic chronic heat stress.

**Figure 1:**
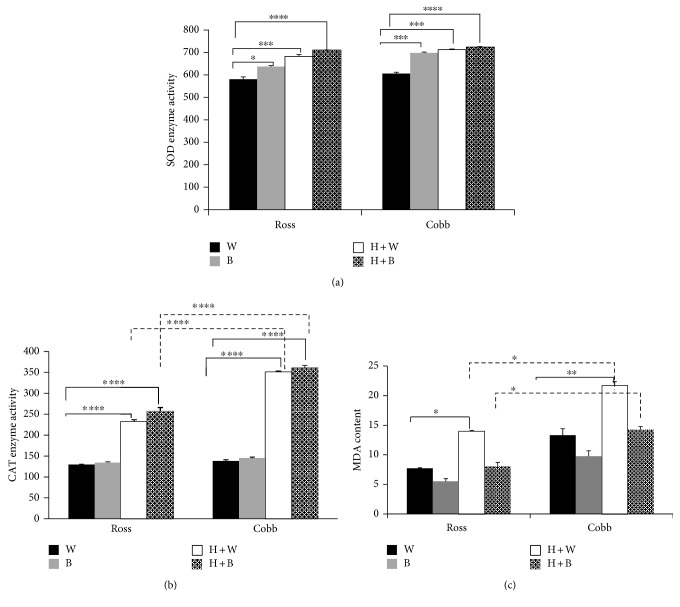
Blue light significantly increases antioxidant enzyme activities and lowers MDA concentration in the liver of two broiler strains (Ross 308 and Cobb 500). Chickens were reared in white light for 3 weeks and after that exposed to cyclic chronic heat stress with white and blue light (H + W and H + B), respectively. Liver samples were collected in sterile PBS for SOD and CAT as well as MDA measurement following the manufacture's protocol. (a) represents SOD enzyme activity. CAT enzyme activity and MDA cellular content were shown in (b) and (c), respectively. Mean ± SEM is shown. ^∗^, ^∗∗^, and ^∗∗∗^ denote statistical significance (two-way ANOVA) with a *p* < 0.05, *p* < 0.01 and *p* < 0.001, respectively.

Figures 1(a) and 1(b) show the effect of strain, light, and strain + light interaction on SOD and CAT enzyme activities in the liver after heat stress, respectively. Light effect on the level of SOD activity was statistically significant (two-way ANOVA, *p* < 0.0001 for light treatment). SOD activity displayed a significant increase in the case of blue light (B), heat stress (H + W), and heat stress with blue light (H + B) compared to white (W) in both Ross and Cobb chickens (two-way ANOVA, in the case of Ross, for B *p* < 0.05; for H + W *p* < 0.001; and for H + B *p* < 0.0001, in the case of Cobb *p* < 0.001 for B, H + W and *p* < 0.0001 for H + B). Besides, CAT enzyme showed a similar response to SOD enzyme activity following heat stress. Strain, light effects, and their interaction were statistically significant (two-way ANOVA, *p* < 0.0001 of the two factors and their interaction). A significant increase of CAT enzyme activity was noticed in the case of heat stress (H + W) and using blue light during heat stress (H + B) compared to white (W) in Ross and Cobb (two-way ANOVA, *p* < 0.0001 for H + W and H + B in the case of Ross and Cobb). Additionally, a significant increase of CAT enzyme activity was found in Cobb compared to Ross chicken at the level of H + W and H + B (two-way ANOVA, *p* < 0.0001 for H + W and H + B) discontinued line in Figure 1(b).

Figure 1(c) shows the MDA contents in Ross and Cobb following heat stress. Strain and light effects on MDA content were statistically significant (two-way ANOVA, *p* < 0.001 for strain and light). Since MDA is an indicator of lipid peroxidation that occurs consequently to heat stress, a significant increase in MDA concentration was detected in the case of H + W for Ross and Cobb compared to white (W) (two-way ANOVA, in the case of Ross, for H + W *p* < 0.05, in the case of Cobb *p* < 0.01 for H + W). Interestingly, using blue light only and during heat stress (B and H + B, resp.) induced a lower concentration of MDA compared to white light (W). Moreover, Ross and Cobb manifested a significantly different response in MDA content, whereas Cobb chicken had a significant higher MDA concentration compared to Ross chicken in the case of heat stress + white light (H + W) and heat stress + blue light (H + B) (two-way ANOVA, *p* < 0.05 for H + W and H + B) discontinued line Figure 1()(c).

### 3.2. Blue Light Lowers the Tissue Damage Induced by Heat Stress in Chicken Liver

**Figure 2:**
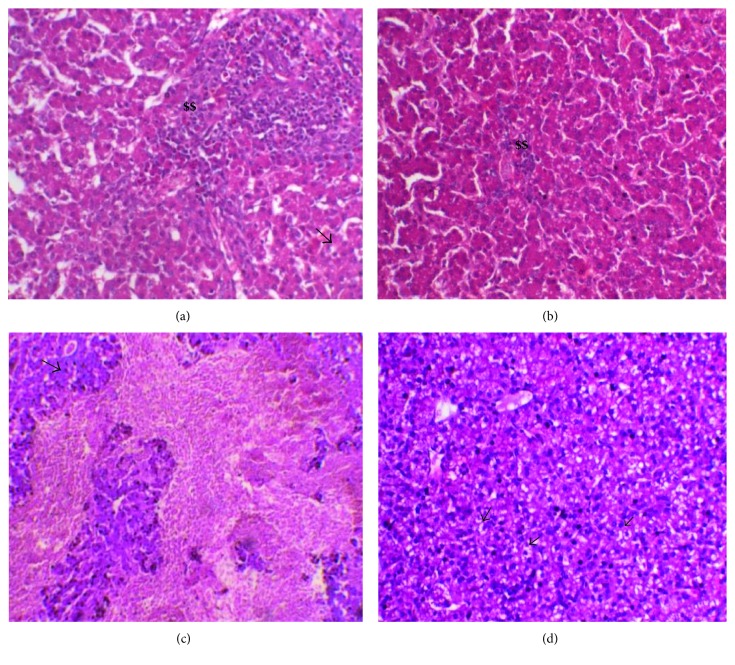
Hematoxylin and eosin staining of liver sections of Ross 308 and Cobb 500: (a) Ross exposed to white light during heat stress H + W and (b) Ross housed with blue light during heat stress H + B. (c, d) The same treatment in the case of Cobb, respectively. $$ refers to mononuclear cell infiltration. Arrows point the fatty changes.

Since high temperature causes severe damage to internal organs' parenchyma such as the liver, we examined the morphological changes in the liver tissue following heat stress in the presence of blue light during treatment. Histological analysis of the liver tissue revealed that heat stress (H + W) in Ross and Cobb induced moderate to severe damage in hepatic tissue. In Cobb, this damage included moderate to severe fatty changes and perivascular mononuclear cell infiltration (in some fields it was admixed with heterophils). Besides, vascular congestion and subcapsular and interstitial hemorrhage were detected (Figure 2(c) as well as in the Supporting information to Figure 2 available online at https://doi.org/10.1155/2017/1351945 H + W for Cobb). For Ross, severe mononuclear cell infiltration admixed with heterophils, hepatic degeneration, and focal necrosis were found (Figure 2(a) as well as in the Supporting information to Figure 2 H + W for Ross). Interestingly, treating heat stress effect using blue light (H + B) lowered the tissue damage caused by heat stress in the two chicken strains. Only mild to moderate vacuolation and mononuclear cell infiltration were found (Figure 2(b) in the case of Ross and Figure 2(d) in the case of Cobb).

### 3.3. Blue Light Significantly Modulates Liver *HSP70*, *HSP90*, *HSF1*, and *HSF3* Gene Expression in Chicken Liver

**Figure 3:**
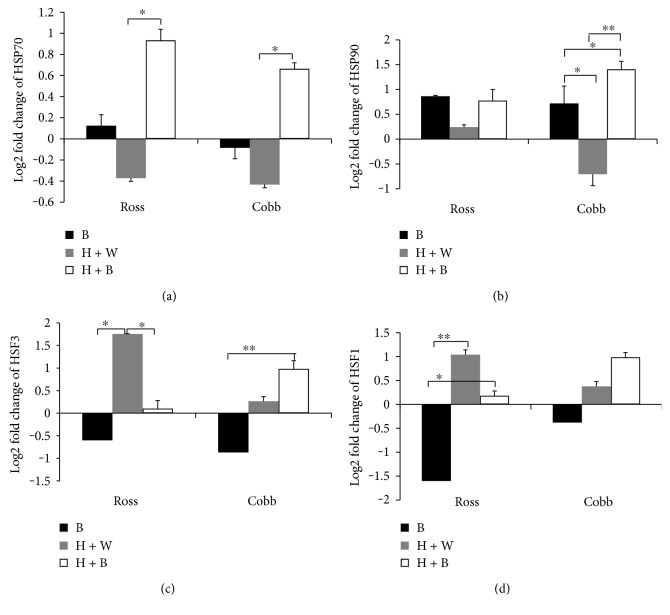
Blue light significantly modulates liver *HSP70*, *HSP90*, *HSF1*, and *HSF3* gene expression in the chicken liver. The relative gene expression levels of *HSP70*, *HSP90*, *HSF3*, and HSF1in the liver of Ross and Cobb exposed to blue light, heat stress in white light, and heat stress in blue light (B, H + W, and H + B, resp.) were measured. The gene expression levels were normalized against control (W) and against two housekeeping genes (*β-*actin and GAPDH). The expression levels were presented as log2 fold change and shown in the figure as mean ± SEM. ^∗^, ^∗∗^, and ^∗∗∗^ denote statistical significance (two-way ANOVA) with a *p* < 0.05, *p* < 0.01, and *p* < 0.001, respectively.

Blue light significantly modulated bird's resistance to heat stress by increasing the level of SOD and CAT. Moreover, it reduced the damaged effect of heat stress on the liver tissue indicated by decreasing MDA concentration in Ross and Cobb (Figure 1() and Figures 2(b) and 2(d), resp.). Therefore, we further examined how blue light regulates the relative mRNA expression of *HSPs* (*HSP70* and *HSP90*) as well as *HSF3* and *HSF1*. The analysis was performed in the liver of Ross and Cobb chickens following heat stress using qPCR. Figures 3(a), 3(b), 3(c), and 3(d) represent the relative gene expression level of *HSP70*, *HSP90*, *HSF3*, and *HSF1*, respectively, from 3 birds for each treatment compared to normal white (W). Blue light, similarly modulated the expression level of *HSP70* and *HSP90* in Ross and Cobb without strain differences (two-way ANOVA, *p* < 0.01 and *p* = 0.05 for blue light effect in the case of *HSP70* and *HSP90*, resp.).

In Ross and Cobb, normally, blue light (B) did not affect much the *HSP70* gene expression where only a slight increase and decrease of *HSP70* gene expression were detected, respectively. However, heat stress caused nonsignificant downregulation of *HSP70* gene expression when used with white light (H + B) in both Ross and Cobb. Nevertheless, when blue light was used during heat stress (H + B), an interesting significant upregulation of *HSP70* gene expression was detected (*p* < 0.05) in the two chicken strains (Figure 3(a)).

For *HSP90* gene (Figure 3(b)), it showed a similar expression pattern to *HSP70* gene. There was no strain differences and only differences due to light treatment were detected without light + strain interaction (two-way ANOVA, *p* > 0.05 for strain and interaction; *p* < 0.05 for light treatment). In Ross, B, H + B, and H + W revealed a nonsignificant upregulation of *HSP90* gene expression level. However, in Cobb, the response to *HSP90* gene was different. Normally and during heat stress, blue light (B and H + B, resp.) induced a significant upregulation of *HSP90* gene (*p* < 0.05; *p* < 0.01, resp.) when compared to heat stress only (H + W) which displayed a downregulation of *HSP90* gene expression level. Additionally, blue light during heat stress stimulated more *HSP90* gene expression compared to under normal condition (B) (*p* < 0.05). These results demonstrate that blue light variably regulates HSP (*HSP70* and *HSP90*) gene expression between the two chicken strains. It induces more *HSP70* and *HSP90* gene expression. Most of the increases in *HSP70* and *HSP90* expression occurred by using blue light during heat stress.

Further, we examined the mRNA expression level of *HSF3* and *HSF1* in the liver of Ross and Cobb chickens. Figures 3(c) and 3(d) show the effect of strain, light, and strain + light interaction on gene expression level of *HSF3* and *HSF1* in the liver of Ross and Cobb chickens, respectively. Statistical analysis revealed a nonsignificant difference due to strain and strain + light interaction. However, light treatment showed a significant effect (two-way ANOVA, *p* > 0.05 for strain and interaction; *p* < 0.05 for light treatment). The expression levels of *HSF3* and *HSF1* genes were similar in Ross. Heat stress (H + W) induced a significant upregulation of *HSF3* and *HSF1* which significantly decreased when the blue light was used during heat stress (*p* < 0.05). Moreover, blue light, alone (B), significantly downregulated the expression levels of *HSF3* and *HSF1* (*p* < 0.01) (Figures 3(c) and 3(d)).

In Cobb, the expression level of the two genes was similar to Ross. Heat stress (H + W) induced a slight increase in the expression level of both *HSF3* and *HSF1*. This effect was significantly increased when blue light was applied during heat stress (H + B). It induced a significant upregulation of *HSF3* and *HSF1* gene expression. However, in the case of using blue light alone (B), it induced a significant downregulation especially for *HSF3* (*p* < 0.01).

In conclusion, using blue light during heat stress regulated the expression levels of *HSP70*, *HSP90*, *HSF1*, and *HSF3* though variably in the different broiler strains. A similar increase in the level of expression of HSPs (*HSP70* and *HSP90*) in Ross and Cobb was found. However, in the case of *HSF1* and *HSF3*, this resulted in an upregulation in their expression levels in Cobb but a downregulation in the case of Ross.

### 3.4. Variations of SOD and CAT Antioxidant Enzyme Activities Could Be Predicted from Their Respective Gene Expression

**Figure 4:**
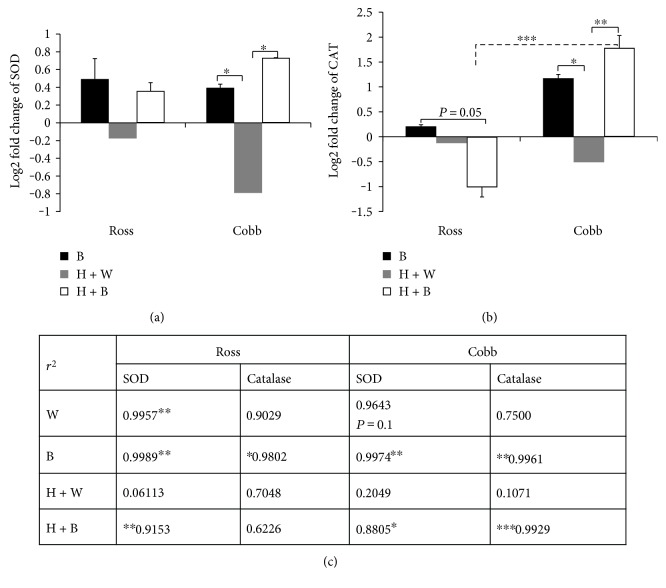
Variations of SOD and CAT antioxidant enzyme activities could be predicted from their respective gene expression. The relative gene expression levels of antioxidant enzymes SOD and CAT in Ross and Cobb in the three groups, blue light (B), heat stressed with white light (H + W), and heat stressed with blue light (H + B) compared to the control group which kept at 24°C and white light. Two housekeeping genes (*β*-actin and GAPDH) were used to normalize the gene expression level. The expression levels were presented as log2 fold change and shown in the figure as mean ± SEM. ^∗^, ^∗∗^, and ^∗∗∗^ in (a) and (b) denote statistical significance (two-way ANOVA) with a *p* < 0.05, *p* < 0.01, and *p* < 0.001, respectively. (c) represents regression analysis of the association between antioxidant enzyme activities and their gene expressions. The analysis was performed at 90% confidence level. The *r*^2^ values are shown. ^∗^, ^∗∗^, and ^∗∗∗^ denote statistical significance at *p* < 0.1, *p* < 0.05, and *p* < 0.01, respectively.

Blue light significantly induced more SOD and CAT enzyme production during heat stress (H + B) (Figures 1(a) and 1(b)). To address how it modulates their gene expression levels, we measured *SOD* and *CAT* mRNA expression levels in the liver of Ross and Cobb chickens. Figures 4(a) and 4(b) show the strain, light treatment, and strain + light interaction effects on *SOD* and *CAT* gene expression levels. For *SOD*, only the light treatment caused significant difference, while in the case of *CAT*, there were significant differences due to strain, light treatment, and strain + light interaction effects (two-way ANOVA, in the case of *SOD* for strain and light + strain interaction *p* > 0.05; for light treatment *p* < 0.05; in the case of *CAT*, strain *p* < 0.01; light treatment *p* = 0.05; and strain + light interaction *p* < 0.01). *SOD* gene expression level, in Ross and Cobb, was upregulated when blue light was used (B and H + B). This upregulation was significant, in case the of Cobb, when compared to heat stress (H + W) which stimulated a significant downregulation (*p* < 0.05).

*CAT* gene reacted differently to heat stress and light treatment between the two chicken strains. For Cobb, it responded similarly to *SOD* gene. Thus, blue light resulted in a significant upregulation either normally (B) or when it was applied during heat stress (*p* < 0.05; *p* < 0.01, resp.) compared to heat stress (H + W) which was characterized by a significant downregulation. However, in the case of Ross, blue light significantly downregulated *CAT* gene expression when used during heat stress (*p* = 0.05). Additionally, there were significant differences in the expression level between Ross and Cobb (*p* < 0.001). In conclusion, blue light regulated *SOD* and *CAT* gene expression levels during heat stress.

To better understand how the variation in the enzymes activities could be predicted from their gene expression, linear regression analysis was performed (Figure 4(c)). For linear regression analysis, the question posed in the test was “what would best predict variations in the level of antioxidant enzymes (SOD and CAT) from their gene expression levels?” The *r*^2^ value, a measurement of the linear relationship between these 2 parameters, and the *p* value set at 90% confidence level are reported.

For Ross, a strong significant association was detected between SOD enzyme and its gene expression level in the case of the control (W), blue light (B), and heat stress + blue light (H + B) (*p* < 0.05 for each). Nevertheless, a very weak nonsignificant association was detected in the case of heat stress (H + W). Additionally, CAT enzyme showed a similar strong significant relationship with its gene expression in the case of blue light (B) (*p* < 0.1). Moreover, a nonsignificant association was detected in the case of the control (W), heat stress (H + W), and heat stress + blue light (H + B).

For Cobb, SOD enzyme exhibited a similar association to its gene, as in the case of Ross. A significant association was detected in the case of the control (W), blue light (B), and heat stress + blue light (H + B) (*p* = 0.1 for W; *p* < 0.0.05 for B; and *p* < 0.1 for H + B). On the other hand, in the case of heat stress (H + W), a weak nonsignificant relationship was found. For CAT, blue light (B and H + B) induced a significant relationship between CAT enzyme and its gene expression (*p* < 0.05 for B; *p* < 0.01 for H + B). However, under normal condition (W) and in the case of heat stress (H + W) nonsignificant association was found.

### 3.5. Blue Light Significantly Regulates Bird's Temperature during Heat Stress Suggesting an Association with Changes in Heat Shock Biomarker Genes

**Figure 5:**
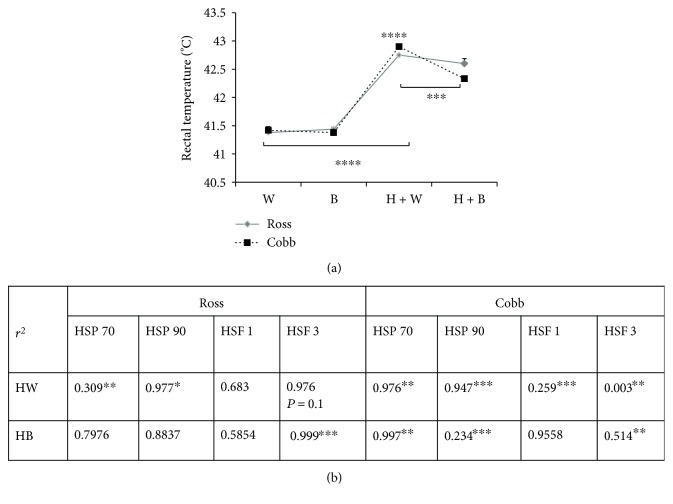
Blue light significantly regulates bird's temperature during heat stress which could be associated with change in heat shock biomarker genes. (a) represents mean ± SEM of the bird's temperature in the case of W, B, H + W, and H + B. ^∗^, ^∗∗^, and ^∗∗∗^ denote statistical significance (two-way ANOVA) with a *p* < 0.05, *p* < 0.01, and *p* < 0.001, respectively. (b) represents regression analysis of the association between the gene expression level (fold change) of heat shock protein and heat shock factor *HSP70*, *HSP90*, *HSF3*, and *HSF1*, respectively, and bird's temperature. Analysis was performed at 90% confidence level. The *r*^2^ values are shown. ^∗^, ^∗∗^, and ^∗∗∗^ denote statistical significance at *p* < 0.1, *p* < 0.05, and *p* < 0.01, respectively.

Figure 5(a) represents strain, light treatment, and strain + light interaction effects on bird's temperature (rectal temperature) during normal condition and heat stress. Strain and strain + light interaction did not show a statistically significant effect on the bird's temperature while light treatment did (two-way ANOVA, for strain and strain + light interaction *p* > 0.05; for light treatment *p* < 0.0001, resp.). Compared to chicken housed under control temperature (W), a significant increase in bird's temperature was recoded due to heat stress regardless of light treatment (H + W and H + B) in both Ross and Cobb (*p* < 0.0001 for each). Interestingly, a significant reduction in the bird's temperature was recorded when blue light was used during heat stress (H + B) compared to heat stress only (H + W). This reduction in body temperature was significant in the case of Cobb compared to Ross which exhibited a nonsignificant reduction in bird's temperature (*p* < 0.001 for Cobb). Moreover, birds reared in blue light during heat stress (H + B) showed less panting, were more relaxed and calm, and exhibited better feed intake in comparison to heat-stressed birds (H + W).

Since heat stress modulated heat shock biomarkers such as *HSP70*, *HSP90*, *HSF3*, and *HSF1* gene expression (Figure 3), a linear regression analysis was performed to examine how the variation in bird's temperature could be explained (Figure 5(b)). We hypothesized that the changes in bird's temperature would best predict variations in the *HSP70*, *HSP90*, *HSF3*, and *HSF1* gene expression levels. The *r*^2^ value and the *p* value, set at 90% confidence level, are reported (Figure 5(b)).

For Ross, heat stress (H + W) induced a strong significant relationship between changes in bird's temperature and *HSP90* and *HSF3* gene expression (*p* < 0.1 and *p* = 0.1, resp.). Additionally, *HSP70* gene expression following heat stress showed a significant moderate correlation with the variation in bird's temperature (*p* < 0.05). However, modulation of *HSF1* gene expression due to heat stress was not significantly related to bird's temperature. Moreover, using blue light during heat stress (H + B) resulted in a significant relationship with *HSF3* gene expression (*p* < 0.01). However, nonsignificant correlation was found in the case of *HSP70*, *HSP90*, and *HSF1* gene expression.

For Cobb chickens, changes in bird's temperature displayed more significant correlations with *HSP70*, *HSP90*, *HSF3*, and *HSF1* gene expression following heat stress in the case of white and blue lighting (H + W and H + B, resp.). *HSP70* gene expression demonstrated a strong significant association with the variations in bird's temperature in the case of ((H + W and H + B); *p* < 0.05 for each). Additionally, a significantly, strong and low correlation was found for *HSP90* gene expression (*p* < 0.01 in the case of H + W and H + B, resp.). Likewise, the variations of *HSF3* gene expression showed very low and moderate significant association with the changes in bird's temperature after heat stress ((H + W and H + B, resp.) (*p* < 0.05 for each)). Also, the changes in *HSF1* gene expression was significantly correlated to body temperature variation after heat stress (H + W); *p* < 0.01).

## 4. Discussion

Several studies addressed the destructive impact of heat stress on poultry, especially chicken, well-being, and production. Heat stress induces oxidative damage which is characterized by production of reactive oxygen species (ROS) in excess of cellular antioxidants [35, 36]. ROS are highly toxic and modify the cellular macromolecules including lipid, protein, and nucleic acid (DNA and RNA) [7]. As a result, their accumulation results in a variety of cellular dysfunctions including lipid peroxidation, protein oxidation, and cell death [35, 37]. Thus, antioxidative enzyme system (including SOD as well as CAT) represents the first line of cellular defense to heat shock. They lower the free radical concentration in the cells by preventing their formation. Additionally, they enhance the mitochondrial electron chain efficiency and diminish the electron leakage resulting in superoxide production. Likewise, they scavenge the initial radicals (such as peroxyl radicals) by stimulating the expression of various transcription factors (e.g., Nrf2 and NF-*κβ*) resulting in preventing the propagation of lipid peroxidation [38–41]. Alteration of these enzyme activities can modify the balance between the production of ROS and the antioxidant system. Consequently, a reduction in animal's productive and reproductive performances and immunity incompetence occurs [42].

In the present study, we addressed the possible regulatory mechanisms on antioxidant enzymes following exposure to blue light under heat stress in two different commercial broiler strains, Ross and Cobb. Ross 308 and Cobb 500 were used in this work because they are the most common commercial broiler strains used for production purpose. Additionally, Cobb 500 had better overall performance than Ross 308 [43, 44]. Moreover, broiler chickens are known to be highly affected by heat stress due to that selection for higher growth rate is associated with decreased resistance to heat stress [16, 45, 46].  Figure 1 represents the effect of blue light on SOD and CAT activities and MDA content following heat stress. In this regard, during heat stress, the elevation in the level of SOD and CAT enzymes is necessary and represents one of the most crucial defense systems of cells to overcome the deleterious effect of HS [47, 48]. This limits the excessive oxidation caused by the accumulation of ROS and thereby protect the cells by maintaining the steady state level of free radicals within the cells [2, 11]. The results in Figure 1 clearly show a striking modulation in the SOD and CAT enzyme activities following exposure to blue light. Interestingly, blue light in the case of B and H + B induced more enzyme activity compared to normal and heat stress, respectively. The most notable is the significantly higher activities of these enzymes following exposure of the birds to blue light during heat stress. Considering that higher level of SOD and CAT enzymes protects against heat stress destructive effects [2], this result is a strong indication of the protective regulatory mechanisms of blue light during heat stress [27]. We suggest that using blue light during heat stress may enhance the bird's resistance to heat stress oxidative injuries to the cells.

On the molecular level, we further examined how exposure to blue light modifies gene expression levels of *SOD* and *CAT* in the two chicken strains (Figure 4). Again, using blue light during heat stress stimulated higher gene expression levels of *SOD* and *CAT* especially in Cobb chicken. However, Ross chicken showed a significant downregulation in the expression of *CAT* gene when blue light replaced the white light during heat stress. The higher gene expression levels in the case of exposure to blue light during heat stress may imply higher enzymatic activity of these antioxidant enzymes. The downregulation of *CAT* gene may explain the significantly lowered CAT enzyme activity of Ross chicken in the case of H + B group compared to Cobb chicken (Figure 1(b)). This was confirmed by using regression analysis that examined the association between antioxidant enzymes and their genes (Figure 4(c)). Significant associations between the enzyme activities and their gene expression demonstrated their tight connection. Our results are in agreement with those results by [49] which showed that the higher antioxidant enzyme activities were due to higher mRNA expression level. The higher expression in the case of H + B represents a protective mechanism of the cells from the negative impact of heat stress [50, 51]. Additionally, the lowered levels of gene expression of *SOD* and *CAT* in the case of heat stress (H + W) agree with results of [52] who reported that the higher temperature during heat stress suppressed the antioxidant activities.

Conversely, for MDA (Figure 1(c)), exposure of birds to blue light during heat stress (H + B) significantly lowered its content compared to heat stress (H + W) which showed a significant increase in MDA content. Higher MDA content is an indicator of lipid peroxidation and consequently more oxidative damage [53–56]. The lowered MDA concentration in the case of H + B suggests a possible role of blue light in lowering the negative effect of heat stress [27].

The effects of exposure to blue lighting on histology of the liver tissue were further examined in Ross and Cobb Figure 2. Heat stress (H + W) induced tissue injuries, infiltration of inflammatory cells, subcapsular and interstitial hemorrhage, hepatic degeneration, and focal necrosis. This was associated with significantly higher H/L ratio in the case of H + W in the two chicken strains (data not shown). Chicken reared under exposure to blue light during heat stress (H + B) showed a reduction in tissue damage effect by heat stress compared to those reared in white light. This was also correlated with lowered H/L ratio in the case of H + B group (data not shown). The blue light effect on the liver tissue concomitant with the lowered MDA concentration (Figure 1(c)) indicates that blue light plays a significant role during heat stress to scavenge the negative effect of heat stress as well as it enhances bird's resistance [23, 26].

Having demonstrated the protective effect of blue light during heat stress by modulating the antioxidant enzyme activity and reducing the liver tissue injuries, we examined its effect on HSPs. In this regard, it is known that one of the cellular consequences of the oxidative stress is protein damage and the subsequent aggregation of unfolded proteins [2]. Heat shock proteins (HSPs) play an essential role in protecting and repairing cells and tissues against stress [12]. They regulate protein processing in the cells and enhance refolding of the damaged protein [57]. The increased expression level of HSPs has been reported to protect against the heat shock adverse subsequent tissue injuries [2, 8, 58–60]. Hence, we examined the possible regulatory impact of blue light on HSP gene expression (Figures 3(a) and 3(b)). Heat stress (H + W) suppressed the expression of *HSP70* and *HSP90* whereas it downregulated *HSP70* and *HSP90* gene expression. The decrease in gene expression was probably due to the severe extent of stress and destructive injuries in the liver [61–63]. This was shown here by the significantly elevated body temperature in the case of (H + W). On the other hand, blue light treatment regulated the level of HSP (*HSP70* and *HSP90*) gene expression. Blue light during heat stress (H + B) induced interesting upregulation of *HSP70* and *HSP90* in the two broiler strains. This effect was tightly associated with the lowered body temperature. Accordingly, HSP upregulation would likely explain the protective effect of the blue light during heat stress.

Since expression of heat shock proteins is regulated by heat shock factor, and HSFs are involved in regulating the cellular response against it, HSF genes (*HSF1* and *HSF3*) were examined (Figures 3(c) and 3(d)). HSF genes were variably expressed during heat stress and in the case of exposure of birds to blue light. In the two chicken strains, heat stress (H + W) induced a significant upregulation of HSF gene expression level. These effects were strongly correlated to higher body temperature in the case of H + W in both strains. HSFs are stress biomarkers and their expression is induced during heat stress [11, 13]. Under normal housing temperature with blue light application (B), the expression level of HSF genes was significantly downregulated. This indicates that blue light exposure does not induce stress on the birds. Likewise, using blue light during heat stress (H + B) led to lower expression levels of *HSF3* and *HSF1* in the Ross strain. This was significantly associated with lowered body temperature, especially for *HSF3*. On the other hand, in the Cobb strain, the expression levels of *HSF1* and *HSF3* genes showed a slight increase when white light was replaced by the blue one during heat stress (H + B). The results of HSP and HSF gene expression levels are consistent with those of [47, 64, 65] who reported that HSFs and HSPs were variably expressed after heat stress. In the latter case, *HSF3* gene expression level continued to increase while that of *HSP 70* decreased. The authors also reported that the expression levels varied depending on the species and the tissue affected.

From the foremost results we reported in this work, replacing white by blue light during heat stress significantly correlated with improved bird's resistance to heat stress and lowered the negative impacts of heat stress. This was clearly shown by reduction of bird's body temperature (Figure 5(a)) in the case of H + B compared to a significant increase in the case of heat stress without blue light exposure (H + W). Cobb chicken displayed a significant decrease in body temperature compared to Ross. This shows that there is a variation in the response to blue light effects between the two strains [55]. This variation in body temperature significantly correlated with the gene expression of heat biomarkers (HSPs and HSFs) (Figure 5(b)).

## 5. Conclusion

Our findings represent the first reported data on the role of monochromatic blue light in regulating the bird's resistance to heat stress. Replacing white light by the blue one during heat stress would modify the heat shock biomarker activities which might enhance the bird's resistance to negative impacts of heat stress. Finally, our results suggest that Cobb 500 have a better response to blue light than Ross 308. Therefore, using blue light during heat stress represents a cheap tool to manage and control heat stress in poultry farms. Therefore, we strongly recommend using blue light in poultry houses during summer.

## Supplementary Material

Supporting information of HSP70, HSP90, HSF3, HSF1, SOD and CAT normal expression. Shown are mean ± SEM of ΔCT values of each gene. ΔCT calculated by subtracting the Ct value of housekeeping genes (Actinβ and GAPDH) from the Ct value of each gene according to Livac method.

## Figures and Tables

**Table 1:** Experimental groups.

Bird strain	Group number	Temp (°C)	Treatment
Ross	W	24	Normal temperature & white light
	B	24	Normal temperature & blue light
	H + W	33 ± 2	Heat stress & white light
	H + B	33 ± 2	Heat stress & blue light
Cobb	W	24	Control & white light
	B	24	Control & blue light
	H + W	33 ± 2	Heat stress & white light
	H + B	33 ± 2	Heat stress & blue light

**Table 2:** Sequence of forward and reverse primers used in real-time PCR.

Gene	Primer	Ref. seq. accession number
*β*-Actin	F: *5′-*ACCTGAGCGCAAGTACTCTGTCT*-3′* R: *5′-CATCGTACTCCTGCTTGCTGAT-3′*	NM_205518.1 [66]
GAPDH	F: *5′-*GGGCACGCCATCACTATCTTC*-3′* R: *5′-*ACCTGCATCTGCCCATTTGAT*-3′*	NM_204305 [67]
HSP70	F: 5-CCAAGAACCAAGTGGCAATGAA*-3′* R: 5-CATACTTGCGGCCGATGAGA*-3′*	EU747335 [49]
HSF1	F: 5-CAGGGAAGCAGTTGGTTCAC TACACG-3 R: 5-CCTTGGGTTTGGGTTGCTCAGTC-3	L06098.1 [66]
HSF3	F: 5-TCCACCTCTCCTCTCGGAAG-3 R: 5-CAACAGGACTGAGGAGCAGG-3	NM_001305041.1 [66]
HSP90	F: 5-GAGTTTGACTGACCCGAGCA*-3′* R: 5-TCCCTATGCCGGTATCCACA*-3′*	NM_206959 [66]
SOD	F: 5-CGGGCCAGTAAAGGTTACTGGAA-3 R: 5-TGTTGTCTCCAAATTCATGCACATG-3	NM_205064.1 [49]
CAT	F: 5-ACTGGTGCTGGCAACCC*-3′* R: 5-ACGTGGCCCAACTGTCAT*-3′*	NM_001031215 [49]

## Abbreviations

CAT: Catalase
HS: Heat stress
HSF: Heat shock factor
HSF1: Heat shock factor 1
HSF3: Heat shock factor 3
HSP: Heat shock protein
HSP70: Heat shock protein 70
HSP90: Heat shock protein 90
MDA: Malondialdehyde
SOD: Superoxide dismutase.
