# Supplementary figures and images for: Modulatory Effect of Monochromatic Blue Light on Heat Stress Response in Commercial Broilers
Source: Oxid Med Cell Longev. 2017 Jun 18;2017:1351945. doi: 10.1155/2017/1351945 (PMC5494062; doi:10.1155/2017/1351945)

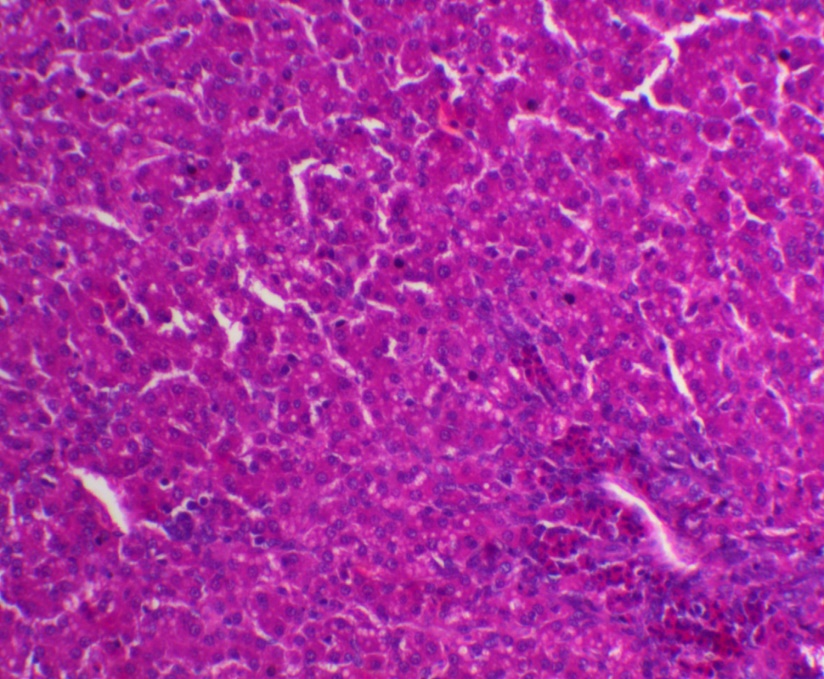

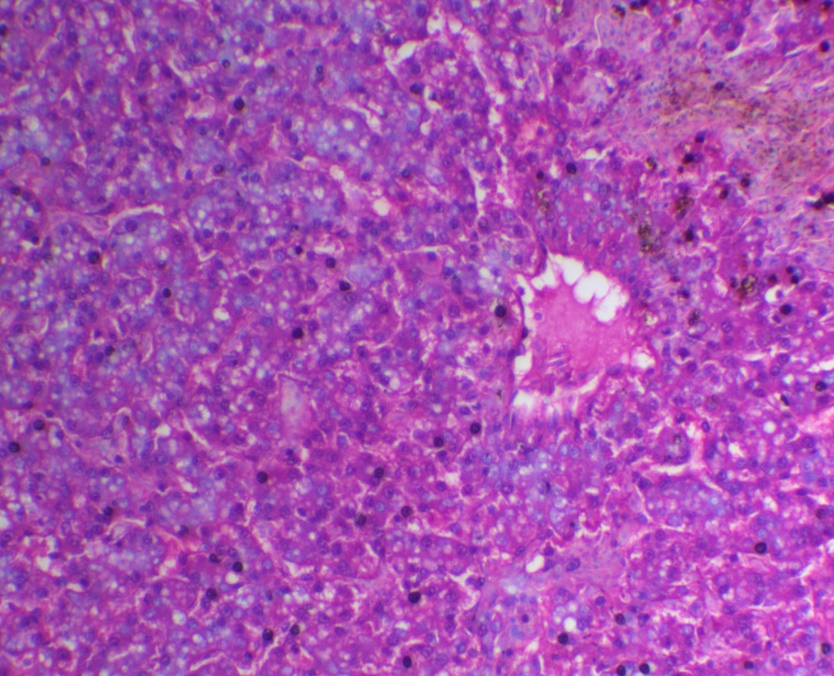

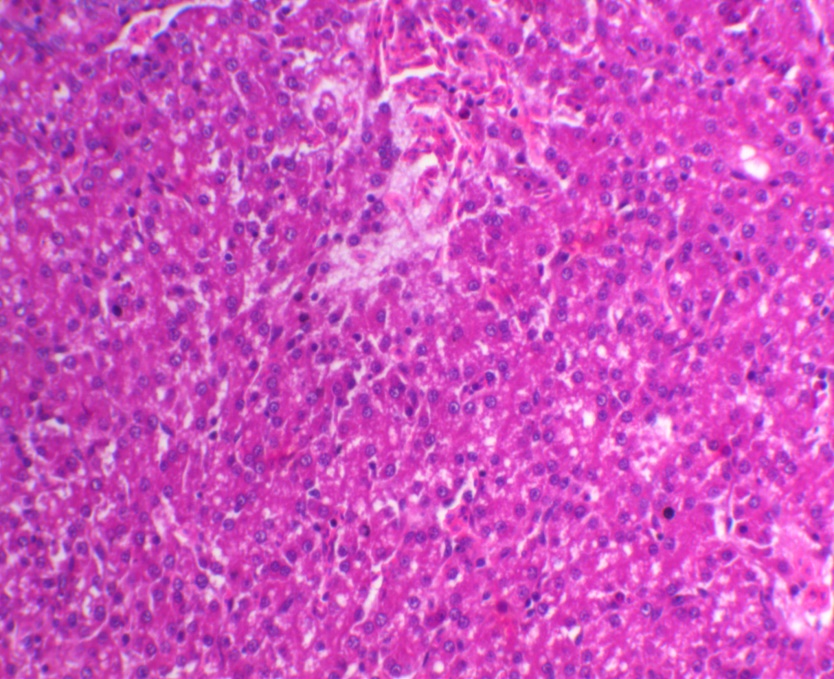

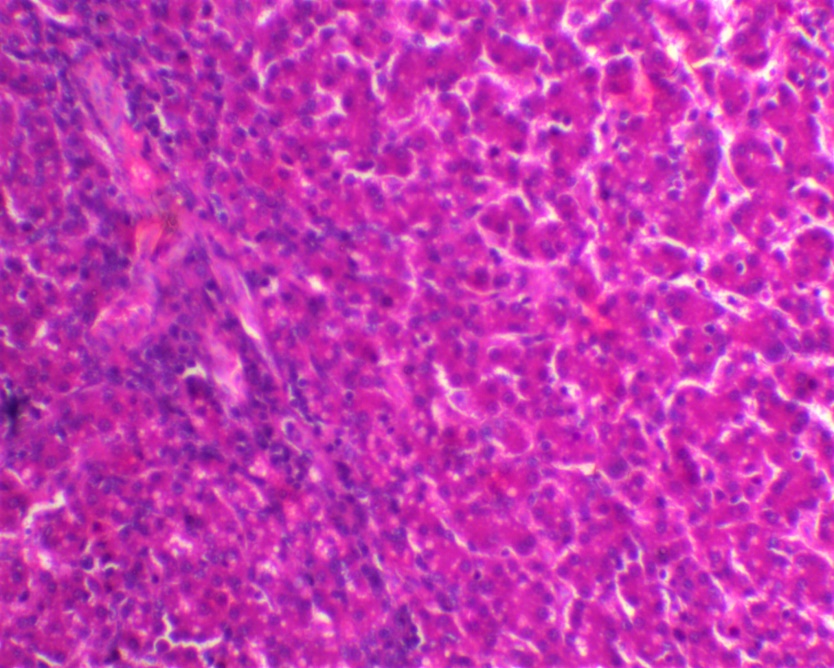


**H+W for Ross**

**H+W for Cobb**

Supplement: Supplementary file 1 — Supporting information of HSP70, HSP90, HSF3, HSF1, SOD and CAT normal expression. Shown are mean ± SEM of ΔCT values of each gene. ΔCT calculated by subtracting the Ct value of housekeeping genes (Actinβ and GAPDH) from the Ct value of each gene according to Livac method. [file 1351945.f1.docx]
