# Supplementary material for: Modulatory Effect of Monochromatic Blue Light on Heat Stress Response in Commercial Broilers
Source: Oxid Med Cell Longev. 2017 Jun 18;2017:1351945. doi: 10.1155/2017/1351945 (PMC5494062; doi:10.1155/2017/1351945)
Supplement: Supplementary file 2 [file 1351945.f2.docx]

**Supporting information of *HSP70, HSP90, HSF3, HSF1, SOD* and *CAT* normal expression.** Shown are mean ± SEM of ∆CT values of each gene. ∆CT calculated by subtracting the Ct value of housekeeping genes (Actinβ and GAPDH) from the Ct value of each gene according to Livac method.
